# Triterpenoid Saponins Isolated from *Platycodon grandiflorum* Inhibit Hepatitis C Virus Replication

**DOI:** 10.1155/2013/560417

**Published:** 2013-12-15

**Authors:** Jong-Woo Kim, Sang Jin Park, Jong Hwan Lim, Jae Won Yang, Jung Cheul Shin, Sang Wook Lee, Joo Won Suh, Soon B. Hwang

**Affiliations:** ^1^B&C Biopharm, Advanced Institutes of Convergence Technology, Suwon 443-270, Republic of Korea; ^2^Division of Bioscience and Bioinformatics, Myongji University, Cheoin-gu, Yongin 449-728, Republic of Korea; ^3^National Research Laboratory of Hepatitis C Virus, Ilsong Institute of Life Science, Hallym University, 1605-4 Gwanyang-dong, Dongan-gu, Anyang 431-060, Republic of Korea

## Abstract

Hepatitis C virus (HCV) infection is a major cause of liver disease, including cirrhosis and hepatocellular carcinoma. Due to significant adverse effects and emergence of resistant strains of currently developed anti-HCV agents, plant extracts have been considered to be potential sources of new bioactive compounds against HCV. The aim of this study was to evaluate the functional effects of triterpenoid saponins contained in the root extract of *Platycodon grandiflorum* (PG) on viral enzyme activities and replication in both HCV replicon cells and cell culture grown HCV- (HCVcc-) infected cells. Inhibitory activities of triterpenoid saponins from PG were verified by NS5B RNA-dependent RNA polymerase assay and were further confirmed in the context of HCV replication. Six triterpenoid saponins (platycodin D, platycodin D_2_, platycodin D_3_, deapioplatycodin D, deapioplatycodin D_2_, and platyconic acid A), PG saponin mixture (PGSM), were identified as active components exerting anti-HCV activity. Importantly, PGSM exerted synergistic anti-HCV activity in combination with either interferon-**α** or NS5A inhibitors. We demonstrated that combinatorial treatment of PGSM and IFN-**α** efficiently suppressed colony formation with significant reduction in drug resistant variant of HCV. These data suggest that triterpenoid saponin may represent a novel anti-HCV therapeutic agent.

## 1. Introduction

Hepatitis C virus (HCV) currently affects nearly 170 million people worldwide, and 3-4 million people are newly infected each year. The majority of these individuals will be chronically infected and may lead to fibrosis, cirrhosis, and hepatocellular carcinoma [1]. HCV is a positive-sense, single-stranded RNA genome of ~9.6 kb. The HCV genome encodes a 3,010-amino-acid protein from a single open reading frame. This polyprotein is processed into structural (core, E1, and E2) and nonstructural proteins (p7, NS2-NS5B). Nonstructural proteins have been attractive to be targets for developing anti-HCV therapy [2–4]. There is no vaccine available for HCV yet. The current standard therapy for chronic HCV, a combination of pegylated interferon- (PEG-IFN-) *α* and ribavirin (RBV), is effective in approximately 70–80% of patients with HCV genotype 2 or 3 infection but effective in less than 50% of those with HCV genotype 1 [5]. Furthermore, therapy with PEG-IFN and ribavirin has serious side effects, including flu-like symptoms, hemolytic anemia, and depression, which often lead to the discontinuance of therapy [6]. Recently, two HCV NS3/4A protease inhibitors, boceprevir (Victrelis) and telaprevir (Incivek), are approved by the U.S. Food and Drug Administration (FDA). However, therapeutic strategy using viral proteins is not fully successful due to error prone nature of HCV RNA replication. For these reasons, there is an urgent need to develop additional therapies that are less toxic, and inexpensive and result in higher sustained virological response (SVR) either as combination or replacement therapies. Discovery of a potent drug candidate from natural products would be useful in overcoming side effects and in generating more synergistic activity.

Platycodi Radix is the root of *Platycodon grandiflorum *(PG). This plant grows wild in east Asian countries and is reported to contain significant amounts of carbohydrate, protein, lipid, and ash. Additionally, PG contains various kinds of triterpenoid saponins. Much attention from pharmaceutical industry has been paid to PG due to its various efficacies on diseases. PG extract has been used as a food additive and oriental herb medicine to cure various diseases, including bronchitis, asthma and pulmonary tuberculosis, and inflammatory diseases. It has been also reported that Platycodi Radix stimulates antioxidants, immunostimulation, and antitumor activity and prevents hyperlipidaemia and obesity [7–9]. Moreover, it has been shown to improve the immunogenicity of virus vaccines in mice by enhancing both humoral and cellular immune responses [10, 11]. Interestingly, the extracts from PG prevented chemical-induced hepatotoxicity [12–14]. Nevertheless, the inhibitory effect of triterpenoid saponin extract from PG on HCV replication has never been explored thus far.

The present study investigated the suppressive activity of root extract from PG against HCV replication using Huh7 cells harboring HCV genotype 1b subgenomic replicon. The active fraction pooled from *Platycodon grandiflorum* saponin mixture (PGSM) was found to have potent anti-HCV activity. We identified 6 triterpenoid saponins (PD, PD_2_, PD_3_, DPD, DPD_2_, and PA) as active components exerting inhibitory activity against HCV replication in subgenomic replicon cells. We further verified antiviral activity of these compounds using RNA-dependent RNA polymerase (RdRp) assay. Furthermore, antiviral properties and synergistic effects of triterpenoid saponins on interferon and other direct acting antiviral (DAA) drugs were verified in HCV replicon cells.

## 2. Materials and Methods

### 2.1. Preparation of Crude Extract and Various Fractions

*Platycodon grandiflorum* was cultivated for three years in Gyeongbuk Province, South Korea. One kg of dried roots of *Platycodon grandiflorum* was cut into slices, and root slices were extracted using 5 L of distilled water at 90°C for 6 h, filtered, and concentrated under reduced pressure to yield a PG extract (85 g). The powder of PG extract was dissolved in distilled water and then subjected to a reverse phase C_18_. The sample was serially eluted with water, 3–5% acetonitrile, 10% methanol, 30% methanol, 50% methanol, 70% methanol, and 100% methanol. The sample isolated in 50% methanol fraction contained triterpenoid saponins and exerted the highest anti-HCV activity and was designated as PGSM. PGSM was further purified by using preparative high-performance liquid chromatography (HPLC) as reported previously [15].

### 2.2. Analysis of PGSM by HPLC/ELSD and LC/MSD

HPLC analysis of PGSM was performed on an Agilent 1100 series HPLC (USA) equipped with a Sedex 55 evaporative light scattering detector (ELSD; SEDERE, Alfortville, France). A sample was separated in a Gemini C_18_ column (100 mm × 4.6 mm, 3 *μ*m particle size; Phenomenex, Torrance, CA, USA) with a precolumn (C_18_, 3.5 *μ*m, 2 × 20 mm) at room temperature. The mobile phase consisted of 0.1% formic acid/methanol/acetonitrile (75 : 5 : 20, v/v/v; A) and 0.1% formic acid/methanol/acetonitrile (70 : 5 : 25, v/v/v; B), and gradient runs were programed as follows: 0–10 min in the absence of B, 10–17 min (0–50% B), 25–34 min (50–80% B), 42–52 min (100% B), and then equilibration in the absence of B for 10 min at a flow rate of 1 mL/min. The injection volume was 20 *μ*L. The ELSD was set to a probe temperature of 70°C, a gain of 7, and the nebulizer gas nitrogen adjusted to 2.5 bar.

The electrospray ionization-mass spectrometric (ESI-MS) analysis was performed on an Agilent 5989 mass spectrometer with an ESI interface fitted with a hexapole ion guide. Chromatographic separation of the PGSM was performed as described above. The optimal condition for the analysis of triterpenoids employed pneumatic nebulization with nitrogen (45 p.s.i.) and a counterflow of nitrogen (9 L/min) heated to 350°C for the nebulization and desolvation of the introduced liquid. Mass spectrometry was performed using the negative ion mode and the scan mode; this process detected from 100 to 2000 *m/z* with a dwell time of 300 ms.

### 2.3. RdRp Assay

Recombinant HCV NS5B polymerase from HCV genotype 1b carrying an N-terminal GST-tag and C-terminal 21-amino acid truncation (NS5BCΔ21) was expressed in *Escherichia coli *and purified as previously described with some modifications [16]. The colorimetric reverse transcriptase assay kit (Roche Applied Science) was applied to perform RdRp assay. The inhibition of HCV RdRp activity was determined by the amount of double-stranded RNA synthesized by the recombinant NS5BΔC21 in the presence of various test compounds using HCV 3′-UTR as a template.

### 2.4. HCV NS3/4A Protease Assay

The NS3/4A serine protease assay was performed using a HCV protease assay kit (SensoLyte, AnaSpec, Fremont, CA) according to the manufacturer's instructions. Briefly, HCV-NS3/4A protease purified from *E. coli* was mixed with the test compound in the assay buffer. After 15 min incubation at room temperature, EDANS/DABCYL-based fluorescence resonance energy transfer (FRET) peptide substrate solution was added and mixed. The FRET substrate was cleaved specifically by HCV NS3/4A protease, thereby liberating the C-terminal peptide-fluorophore fragment from the proximity quenching effect of the dark quencher, resulting in increase of fluorescence. The fluorescence intensity was measured immediately and continuously at excitation/emission at 340 nm and 490 nm.

### 2.5. *In Vitro* Anti-HCV Assay in HCV Replicon Cells

Huh7 cells harboring HCV subgenomic replicon (genotype 1b) [17] were maintained in the presence of 0.25 mg/mL G418 (Invitrogen, Carlsbad, CA). HCV replicon cells were seeded at a density of 1 × 10^4^ cells/well in a 96-well plate and incubated at 37°C and 5% CO_2_. Following 24 h incubation, the culture medium was replaced with a medium containing serially diluted test compounds in the presence of 2% FBS and 1% DMSO. After cells were treated for 72 h with PGMS and triterpenoid saponins, total RNAs were extracted using a CellAmp Direct RNA Prep Kit (Korea Biomedical, Seoul, Korea). The HCV RNA levels were quantified by a quantitative real-time polymerase chain reaction (qRT-PCR) assay using IQ5 real-time PCR detection system (Bio-Rad, Hercules, CA, USA) with HCV-specific primers (5′-GAC ACT CCA CCA TAG ATC ACT C-3′ and 5′-CCC AAC ACT ACT CGG CTA G-3′) and probe (5′-FAM-CCC AAA TCT CCA GGC ATT GAG CGG-3′ BHQ-1). Results were normalized to glyceraldehyde-3-phosphate dehydrogenase gene (GAPDH). Anti-HCV activity was determined by HCV RNA levels in compound-treated cells as compared to mock-treated cells.

### 2.6. *In Vitro* Anti-HCV Assay in HCV-Infected Cells

Infectious JFH1 viruses (HCV genotype 2a) were generated as described previously [18]. Huh7 cells were seeded in 24-well plates at a density of 5 × 10^4^ cells per well. At 24 h after plating, cells were infected with JFH1 viruses for 2 h. After incubation, the supernatants were replaced with fresh medium. Increasing concentrations of PGMS and its triterpenoid saponins were added in a medium containing 2% DMSO, and cells were incubated at 37°C. At 48 h after infection, total RNAs were extracted and the HCV RNA levels were quantified by qRT-PCR assay. Anti-HCV activity was determined by HCV RNA levels in compound-treated cells as compared to mock-treated cells.

### 2.7. Western Blotting

Total cell lysates were separated by SDS-PAGE and electrotransferred to a PVDF membrane (Bio-Rad). The membrane was blocked with 5% skim milk in Tris-buffered saline (TBS) and incubated with an anti-HCV NS5A monoclonal antibody (Santa Cruz Biotechnology). Proteins were detected using an enhanced chemiluminescence detection system (Intron Biotechnology).

### 2.8. Cell Viability Assay

Cell viability was determined by the colorimetric 3-(4,5-dimethylthiazol-2-yl)-2,5-diphenyltetrazolium bromide (MTT) reagent (Promega Corporation) according to the manufacturer's instructions. The absorbance was detected at 570 nm using a Bio-Tek plate reader (Bio-Tek instrument, Weymouth, MA).

### 2.9. *In Vitro* Combination Studies

HCV Replicon cells were treated with various concentrations of inhibitors in twofold serial dilutions at a final dimethyl sulfoxide concentration of 1% including titrations of each of the compounds alone as reference controls. The concentration used for PGSM ranged from 5 ug/mL to 0.16 ug/mL; for interferon-*α*, the concentration used ranged from 80 U/mL to 2.5 U/mL; the concentration used for R7227 ranged from 2.5 ng/mL to 0.08 ng/mL; and for BMS 790052, the concentration used ranged from 20 pg/mL to 0.63 pg/mL. At three days after treatments, intracellular HCV RNA levels were determined by qRT-PCR. For the Loewe additive model, the experimental data were analyzed by using CalcuSyn (Biosoft, Ferguson, Mo.), a computer program based on the method of Chou and Talalay [19, 20]. A combination index (CI) value for each experimental combination was calculated by a quantitative measure of the degree of drug interaction in terms of additive effect (CI = 1), synergism (CI < 1), or antagonism (CI > 1) for a given endpoint of the effect measurement.

### 2.10. Anti-HCV Assay for 14 Days

HCV replicon cells were plated at 2 × 10^5^ cells per well in a 6-well plate. Each compound was serially diluted in DMEM containing 10% FBS and 0.2% DMSO as reported previously [21, 22]. Culture media were replaced with fresh medium and compounds every 3 days. Cells were harvested at 3, 6, 9, and 14 days and intracellular HCV RNA levels were analyzed by qRT-PCR. The relative copy number of HCV RNA per cell was calculated by comparing RNAs in cells treated with each compound with those RNAs in cells treated with 0.2% DMSO.

### 2.11. Colony Forming Assays

Huh7 cells harboring HCV subgenomic replicon (genotype 1b) were treated with either various concentrations of PGSM alone or in combination with IFN-*α*, BMS790052 (NS5Ai), and VX-950 (PI), respectively. G418 was included in the media to provide selective pressure on HCV replicon cells. After three weeks of treatment, surviving cells were stained with crystal violet [23]. HCV RNAs isolated from colonies were also subjected to sequence analysis.

## 3. Results 

### 3.1. Identification of Active Components Bearing Anti-HCV Activity in the Root of PG

**Figure 1:**
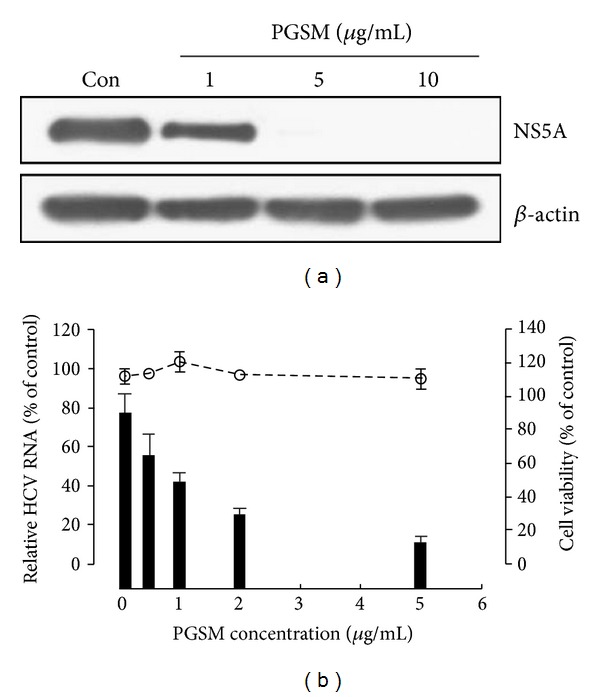
Effect of PGSM on HCV protein expression and RNA replication in HCV replicon cells. Huh7 cells harboring HCV replicon were treated with different concentrations of PGSM for 3 days. 1% DMSO was used as a control (vehicle). (a) Total cell lysates were immunoblotted with an anti-HCV NS5A antibody and anti-actin antibody, respectively. (b) Total RNAs were extracted from cells at 72 h after PGSM treatment, and intracellular HCV RNAs were quantified by qPCR. Relative HCV RNA levels were normalized by cellular GAPDH mRNA. Cell viability was assessed by the MTT assay.

**Figure 2:**
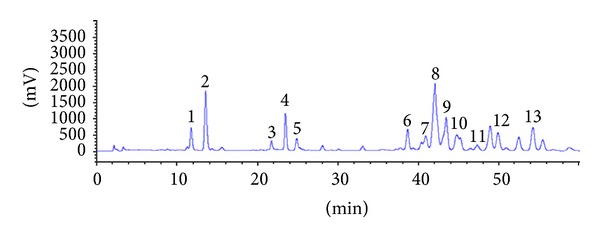
Representative HPLC/ELSD chromatograms of triterpenoid saponins in PGSM. The numbers indicate each triterpenoid saponin: 1, deapi-platycoside E; 2, platycoside E; 3, deapi-platycodin D_3_; 4, platycodin D_3_; 5, platyconic acid A; 6, deapi-platycodin D; 7, platycodin D_2_; 8, platycodin D; 9, polygalacin D; 10, 3′′-O-acetylpolygalacin D; 11, platycodin A. 12, deapio-3′′-acetyl polygalacin D; 13, 2′′-O-acetyl platycodin D.

**Figure 3:**
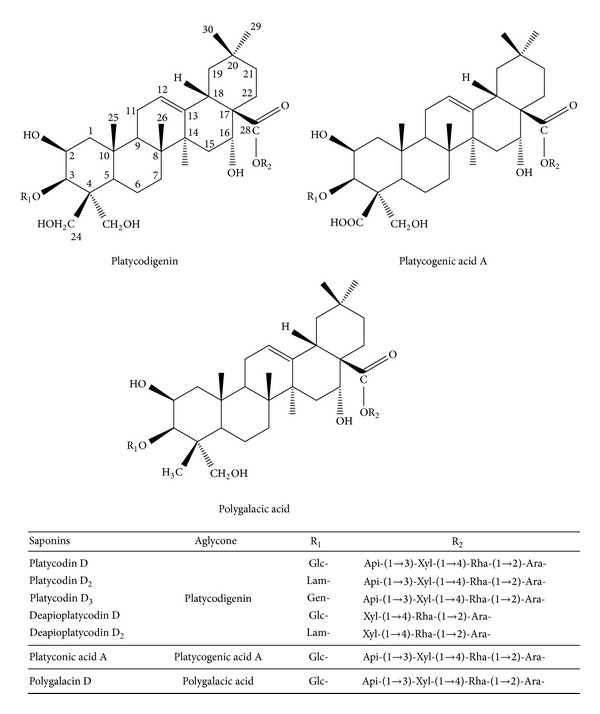
Chemical structures of triterpenoid saponins from the root of PG. Glc, *β*-D-glucopyranosyl; Lam, laminaribosyl; Gen, gentiobiosyl; Api, *β*-D-apiofuranosyl Xyl, *β*-D-xylopyranosyl; Rha, *α*-L-rhamnopyranosyl; Ara, *α*-L-arabinofuranosyl.

PG-extract exerted anti-HCV activity in a dose-dependent manner in HCV subgenomic replicon cells. The half maximal effective concentration (EC_50_) of PGSM to inhibit HCV replication was 35 *μ*g/mL. PG-extract was further fractioned by reverse phase C_18_ open column chromatography and eluted with water (3.41 g), 3–5% acetonitrile (0.52 g), 10% methanol (12.8 g), 30% methanol (2.12 g), 50% methanol (0.22 g), 70% methanol (0.07 g), and 100% methanol (0.04 g), respectively (data not shown). Based on bioactivity-guided screening, HCV subgenomic replicon (genotype 1b) cells were used to assess the anti-HCV activities of various fractions isolated from PG-extract. HCV replicon cells were treated with various fractions using fixed concentration of 10 *μ*g/mL for three days. The fraction eluted with 50% methanol (PGSM) showed a potent anti-HCV activity (EC_50_ = 0.78 *μ*g/mL) as compared to other fractions. To further verify the anti-HCV activity, HCV replicon cells were incubated with various concentrations of PGSM. Cell lysates harvested at two days after PGSM treatment were immunoblotted with anti-NS5A antibody. As shown in Figure 1(a), HCV protein expression was completely inhibited by 5 *μ*g of PGSM. We demonstrated that intracellular HCV RNA levels were significantly reduced by treatment of 2 *μ*g of PGSM (Figure 1(b)). We also showed that treatment of HCV replicon cells with PGSM at concentrations as high as 5 *μ*g/mL induced no cell toxicity as measured by an MTT assay (Figure 1(b)). HPLC/ELSD and LC/MS analysis data showed that PGSM was found in triterpenoid saponin-rich fraction (Figure 2). Triterpenoid saponin-rich fraction was further fractionated into six groups of triterpenoid saponin, including platycodin D (PD), platycodin D_2_ (PD_2_), platycodin D_3_ (PD_3_), deapioplatycodin D (DPD), deapioplatycodin D_2_ (DPD_2_), and platyconic acid (PA) (Figure 3). The purities were ≥95% for the different saponin compounds. The structure of each compound was analyzed by IR, NMR, and MS.

### 3.2. Triterpenoid Saponin Extracts Exerted Anti-RdRp Activity

**Figure 4:**
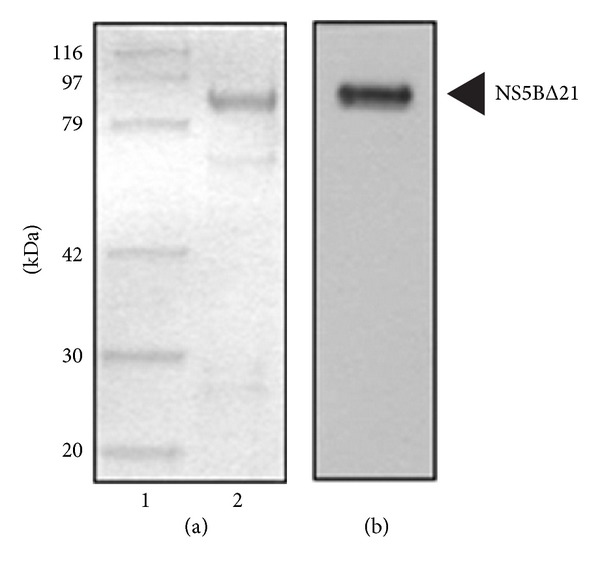
Purification of HCV NS5B protein. (a) HCV NS5B protein (1 *μ*g) eluted from glutathione sepharose affinity column was subjected to SDS-polyacrylamide gel electrophoresis and visualized by Coomassie Brilliant Blue R-250; (b) NS5B protein purified from figure legend to A was immunoblotted with an anti-NS5B monoclonal antibody and visualized with the enhanced chemiluminescence detection. The arrowhead indicates the NS5B protein.

To investigate whether triterpenoid saponins had anti-HCV activity, RdRp assay was performed using recombinant HCV NS5B protein and HCV 3′ UTR as a RNA template. As shown in Figure 4(a), N-terminally truncated (21 aa deletion) NS5B protein was expressed as a GST-tagged protein and further purified as approximately 87 kDa. This protein was verified by immunoblot analysis using anti-HCV NS5B antibody (Figure 4(b)). We showed that all triterpenoid saponins, including PGSM, PD, PD_2_, PD_3_, DPD, DPD_2_, and PA inhibited RdRp activity, and inhibition occurred in a dose-dependent manner with IC_50_ value of 5 *μ*g/mL, 5 *μ*g/mL, 6 *μ*g/mL, 8 *μ*g/mL, 7 *μ*g/mL, 10 *μ*g/mL, and 15 *μ*g/mL, respectively (Table 1). However, these triterpenoid a sponins showed no inhibitory activities on HCV NS3/4A protease in FRET assays (data not shown).

### 3.3. Triterpenoid Saponins Suppress RNA Replication in HCV Replicon Cells and in HCV-Infected Cells

**Figure 5:**
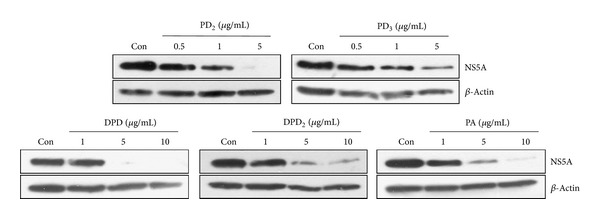
Inhibition of HCV protein expression by triterpenoid saponins in HCV replicon cells. HCV replicon cells were treated with the indicated amounts of triterpenoid saponins. Con indicates a vehicle (DMSO). Three days after treatments, cell lysates were immunoblotted with an anti-NS5A antibody. Actin protein was used as a loading control.

To further verify the antiviral activity of triterpenoid saponins in the context of HCV replication, the effect of triterpenoid saponins on HCV RNA replication was assessed in both Huh7 cells harboring HCV subgenomic replicon and HCVcc-infected Huh7 cells. As shown in Table 2, PGSM and its subfractions of triterpenoid saponins inhibited HCV RNA replication in HCV replicon cells and in HCVcc-infected cells. This inhibition occurred in a dose-dependent manner (data not shown). The EC_50_ values of triterpenoid saponins for RNA replication in HCV subgenomic replicon cells ranged from 0.35 to 2.45 *μ*g/mL. It was noteworthy that the EC_50_ values of HCVcc-infected cells were approximately ten times higher than those of HCV replicon cells (Table 2). We further showed that CC_50_ values of the triterpenoid saponins in Huh7 cells were very high as compared to EC_50_ values, which indicate that these compounds were not cytotoxic at inhibitory concentrations. To further investigate the effect of triterpenoid saponins on HCV protein expression level, HCV replicon cells were treated with the indicated amounts of triterpenoid saponins, and HCV protein expression was detected by immunoblotting with anti-NS5A antibody. As demonstrated in Figure 5, triterpenoid saponins prominently inhibited the HCV protein expression level. Of note, PD_2_ and PD_3_ appear to be more potent than other triterpenoid saponins in anti-HCV activity.

### 3.4. Synergistic Effect of PGSM on IFN-*α* and HCV Inhibitor-Mediated Antiviral Activity in HCV Replicon Cells

The current standard of care for the treatment of chronic HCV is the combination of pegylated IFN-*α* and ribavirin. Combination therapy with two or three drugs which have different modes of action is regarded as a promising way to enhance SVR more than 90% and to suppress mutant strains. The antiviral activities of PGSM in combination with either IFN-*α*, the HCV NS5A inhibitor Daclatasvir (BMS 790052) [24], and the NS3/4A protease inhibitor Danoprevir (ITMN-191(R7227)) [25] were examined in HCV subgenomic replicon cells. Replicon cells were incubated with PGSM in combination with IFN-*α*, BMS-790052, and R7227 at various concentrations and the anti-HCV activity and cytotoxicity were examined as described in Materials and Methods. The combination index (CI) values were analyzed using CalcuSyn software to examine whether the effect of the combination was additive or synergistic. A CI value of 1 indicates an additive effect, a CI value of less than 1 indicates a synergistic effect, and a CI value of greater than 1 indicates antagonism. As shown in Table 3, most CI values were significantly less than 1 (0.34–0.72) when cells were treated with IFN-*α* in combination with various inhibitors. These results indicate that combinations of PGSM with other DAAs have synergistic effect on inhibition of HCV RNA replication in the replicon cells. There was no significant increase in cytotoxicity when PGSM was treated in combination with other DAAs (data not shown).

### 3.5. PGSM Potentiates IFN-*α*-Mediated Anti-HCV Activity

**Figure 6:**
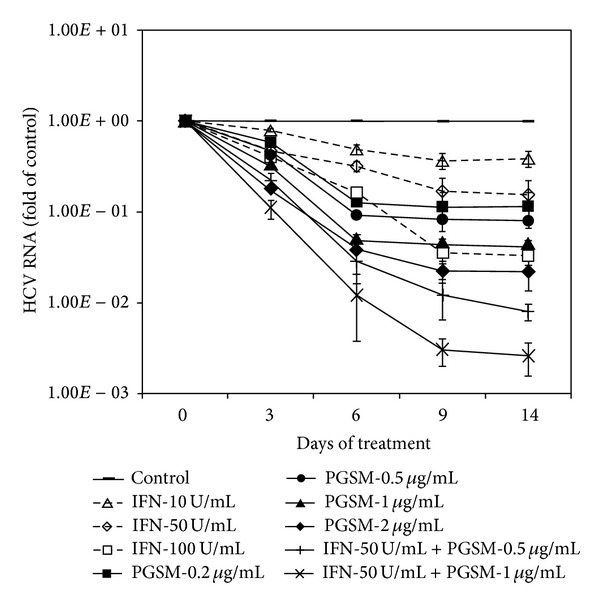
Synergistic effect of PGSM on IFN-*α*-induced anti-HCV activity in HCV replicon cells. HCV subgenomic replicon cells (genotype 1b) were treated with the indicated amounts of either PGSM or IFN-*α* alone or both as indicated. Culture media containing fresh compounds were replaced every three days. At the indicated time points after treatments, intracellular HCV RNA levels were determined by qRT-PCR. The copy number of HCV RNA was calculated from cells treated with compound as compared to that for control. Control indicates that cells were treated with 0.2% DMSO (vehicle).

To investigate whether PGSM was able to induce a multilog reduction of RNA replication in HCV replicon cells, we assessed PGSM activity for 14 days. As shown in Figure 6, PGSM continuously reduced HCV RNA levels in a time- and concentration-dependent manner. At 14 days after treatments of PGSM with 0.2 ug/mL, 0.5 ug/mL, 1.0 ug/mL, and 2.0 ug/mL reduced HCV RNA levels by 0.9 log_10_, 1.1 log_10,_ 1.3 log_10_, and 1.7 log_10_, respectively. Likewise, treatment of IFN-*α* with 10 U/mL, 50 U/mL, and 100 U/mL inhibited HCV RNA levels by 0.4 log_10_, 0.8 log_10_, and 1.5 log_10_, respectively. It was noteworthy that combinatorial treatments of PGSM and IFN-*α* resulted in significant reduction of HCV RNA levels as compared to either PGSM or IFN-*α* alone, suggesting that PGSM may be a potent therapeutic agent for HCV in combination with IFN-*α*.

### 3.6. PGSM Potentiates Colony Suppression in Replicon Cells in Combination with Either IFN-*α* or DAAs

**Figure 7:**
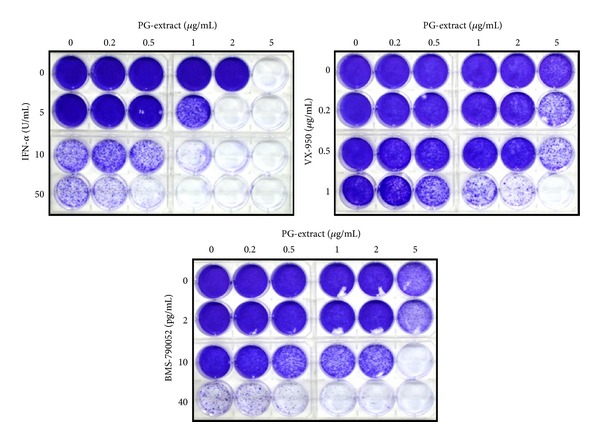
PGSM potentiates colony suppression in replicon cells in combination with IFN-*α* and DAAs. HCV replicon cells were treated with various concentrations of PGSM and IFN-*α*, PGSM and BMS-790052 (NS5Ai), and PGSM and VX-950 (PI) as indicated. Three weeks after treatment, remaining surviving cells were stained with crystal violet.

To further verify the antiviral activity of PGSM in HCV replicon cells, we performed HCV colony forming assay using PGSM with either IFN-*α* or two DAAs, BMS790052 (NS5Ai), and VX-950 (PI). As shown in Figure 7, 5 U/mL of IFN-*α* was unable to inhibit colony formation of HCV replicons. However, cotreatment of 1 *μ*g of PGSM and 5 U/mL of IFN-*α* efficiently suppressed the colony formation, indicating that PGSM potentiates IFN-*α*-mediated anticolony formation. Similar activities of PGSM were observed in both DAA-treated cells (Figure 7). We further showed that the emergence of drug resistant variant was significantly reduced when IFN-*α* or DAAs was cotreated with PGSM in replicon cells (data not shown).

## 4. Discussion

HCV is a common infectious agent affecting approximately 170 million individuals worldwide [26]. Currently, there is no protective vaccine available for HCV. Although current standard therapy, combination of PEG-IFN-*α* and RBV, often showed high SVR in certain genotypes, this therapy accompanies significant adverse effects. The recent approval of the first HCV-specific DAAs that was given in a triple combination with PEG-IFN-*α*/RBV has increased cure rates in genotype 1 naïve patients from ~55% to 75%, at least under conditions of standardized clinical trials, but they still have limitations in the possible dose-limiting adverse effects and low genetic barrier to resistance.

Natural products could be important sources for anti-HCV agents [27]. A variety of medicinal herbs were used to treat HCV, especially for patients who are not eligible for IFN/RBV or who fail to respond to IFN [28]. In the United States, milk thistle and glycyrrhizin are the most popular herbal medicines for the treatment of HCV, and they are often used as adjuncts to conventional therapies. Accumulating pieces of evidence have shown that natural products derived from plants exerted inhibitory effects on HCV replication [29, 30]. For example, Silibin-related flavoligands exhibited an inhibitory effect on HCV RdRp activity and multiple hepatoprotective functions [31, 32]. Epigallocatechin gallate (EGCG), a major component of catechin in tea and certain plants, was shown to have anti-HCV NS5B activity [33]. It has been previously reported that 2-arylbenzofuran derivatives from Mori Cortex Radicis possessed anti-HCV activity [34]. Overall, natural products could be alternative sources to control HCV propagation.

In this study, we demonstrated that the root extracts of PG exhibited inhibitory activity against HCV RNA replication in HCV subgenomic replicon cells. Furthermore, we showed that triterpenoid mixture fraction exerted inhibitory activity against HCV RNA replication. We further identified that PD, PD_2_, PD_3_, DPD, DPD_2_, and PA were the active components in triterpenoid mixture. The EC_50_ values of active saponin against anti-HCV activity in HCV subgenomic replicon cells ranged from 0.35 to 2.45 ug/mL. In fact, all of these active compounds exerted direct anti-NS5B polymerase activity. The IC_50_ values of active saponins for RdRp activity were around 5–15 ug/mL. Of note, these triterpenoid saponins did not show any inhibitory effect on NS3 protease. Moreover, anti-HCV activities of triterpenoid saponins were more potent in the context of HCV replication than *in vitro* enzyme assay system (Tables 1 and 2). These data suggest that either cellular factors or cellular immune responses may be involved in triterpenoid saponin-mediated anti-HCV activity. Further studies are required to elucidate the mechanisms of triterpenoid saponin-induced anti-HCV activity.

We then asked whether IC_50_ in a *μ*g/mL range could be reached in patients. In our animal studies, the half-life of these compounds was 6.57 ± 0.7 h which may be enough to reach the therapeutic concentration in rats. Pharmacokinetic studies showed that the absorption rate was increased 6~10 times higher in intraduodenum (ID) and intraileum (IL) than in oral (Oral-PO) (data not shown). These results suggest that therapeutic concentration can be reached in patients if these compounds are coated for enteric absorption. In fact, preliminary coated PGSM study showed that HCV titers were decreased ≥2 log when was administered to for chronic HCV patients 8 weeks.

Triterpenoid saponins are secondary metabolites of glycosidic nature that are widely distributed in higher plants and are also found in marine invertebrates. Saponins exert a wide range of pharmacological activities, including expectorant, anti-inflammatory, vasoprotective, hypocholesterolemic, immunomodulatory, hypoglycemic, molluscicidal, antifungal, and antiparasitic functions [35]. More than 20 triterpenoid saponins have been isolated from PG. PD and PD_2_ have shown the most potent biological activities among platycodin saponins. It has been reported that PD and PD_2_ are potentially less hemolytic saponin adjuvant eliciting Th1 and Th2 immune responses [10, 36]. PD is also a potent adjuvant of specific cellular and humoral immune responses against recombinant hepatitis B antigen [37]. It has been shown that PD induces apoptosis and decreases telomerase activity in human leukemia cells [38]. PD and 2′′-O-acetyl-polygalacin D2 protect against ischemia/reperfusion injury in the gerbil hippocampus [39]. Saponins from PD also protect against carbon tetrachloride induced hepatotoxicity and against acute ethanol-induced hepatotoxicity in mice [40, 41].

In the present study, we investigated whether combination therapy of PGSM and IFN-*α*, Daclatasvir (BMS 790052, NS5A inhibitor), and Danoprevir (ITMN-191, R7227) would enhance the anti-HCV activity in HCV replicon cells. As shown in Table 3, the highest combination index (CI) values were significantly less than 1 (0.34–0.72) when PGSM was treated in combination with various inhibitors in HCV replicon cells. We demonstrated that treatment of HCV replicon cells with PGSM for two weeks resulted in multilog reduction in HCV RNA levels in a time- and dose-dependent manner. We further showed that PGSM showed a synergistic effect on IFN-*α*-, BMS 790052-, and ITMN-19-induced anti-HCV activity (Figure 4). These data strongly indicate that PGSM may be used as a new regimen in combination with IFN-*α* for treatment of chronic HCV patients. Collectively, we demonstrated for the first time that the triterpenoid saponins from PG extracts exerted suppressive activity on HCV replication. Specifically, inhibitory functions of these saponins on HCV RdRp activity could partly explain the antiviral mechanism in HCV replicating cells. Further studies are necessary to elucidate the mechanism that how triterpenoid saponins inhibit RdRp activity.

Finally, we noticed that therapeutic window for most of the saponins in genotype 2a was rather small because all selectivity indeices (SI) were smaller than 20. Although therapeutic index for the saponins in genotype 1b was higher than genotype 2a, we still need to improve therapeutic window with forthcoming studies. Nevertheless, since the combination treatments of saponins and IFN-*α* and other DAAs showed synergistic effects on anti-HCV activity, saponin may be used as an effective therapeutic agent for certain HCV patients. Taken together, our data indicate that triterpenoid saponin may represent a novel anti-HCV therapeutic agent to control HCV replication.

## Figures and Tables

**Table 1:** Specification of chemical structure and anti-HCV activities of triterpenoid saponin extracts of *Platycodon grandiflorum* on NS5B RdRp and NS3/4A protease functions.

Compounds	Aglycone	Composition of sugar		HCV enzyme (IC_50_, *μ*g/mL)	
		C-3	C-28	HCV NS5B	HCV NS3/4A
PG-extract	—	—	—	67	>100
PGSM	—	—	—	5	>100
Platycodin D	Platycodigenin	Glc-	Api-(1→3)-Xyl-(1→4)-Rha-(1→2)-Ara-	5	>100
Platycodin D_2_		Glc-(1→3)-Glc-	Api-(1→3)-Xyl-(1→4)-Rha-(1→2)-Ara-	6	>100
Platycodin D_3_		Glc-(1→6)-Glc-	Api-(1→3)-Xyl-(1→4)-Rha-(1→2)-Ara-	8	>100
Deapioplatycodin D		Glc-	Xyl-(1→4)-Rha-(1→2)-Ara-	7	>100
Deapioplatycodin D_2_		Glc-(1→3)-Glc-	Xyl-(1→4)-Rha-(1→2)-Ara-	10	>100
Platyconic acid A	Platycogenic acid A	Glc-	Api-(1→3)-Xyl-(1→4)-Rha-(1→2)-Ara-	15	>100

**Table 2:** Inhibitory effects of triterpenoid saponins on HCV replication in HCV replicon cells and in HCVcc-infected cells.

Compounds	Inhibition of HCV RNA replication (EC_50_, *μ*g/mL)		Cytotoxicity (CC_50_, *μ*g/mL)
	CON1 (genotype 1b)	JHF-1 (genotype 2a)	
PG-extract	35		>100
PGSM	0.78	8	25
Platycodin D	0.35	3	25
Platycodin D_2_	1.05	12	25
Platycodin D_3_	2.33	27	100
Deapioplatycodin D	1.2	7	25
Deapioplatycodin D_2_	0.89	8	100
Platyconic acid A	2.45	22	100

**Table 3:** Synergistic effect of PGSM on IFN-*α*-, R7227-, and BMS790052-mediated anti-HCV activity.

Combination with	Combination Index*			Effect
	At EC_50_	At EC_75_	At EC_90_	
**IFN-** **α**	0.45	0.39	0.34	Synergistic
**R7227** (NS3 protease inhibitor )	0.72	0.71	0.70	Synergistic
**BMS790052** (NS5A inhibitor)	0.41	0.42	0.44	Synergistic

HCV replicon cells (genotype 1b) were treated in combination of PGSM and IFN-*α*, R7227, and BMS790052. At 72 h after treatment, anti-HCV activity was determined by qRT-PCR (Taqman). CI values at 50% effective concentration (EC_50_), 75% effective concentration (EC_75_), and 90% effective concentration (EC_90_) were calculated using CalcuSyn software.

*A CI value of 1 indicates additive effect; a CI value of less than 1 indicates synergistic effect; a CI value of greater than 1 indicates an antagonistic effect.

## Abbreviations

HCV: Hepatitis C virus
HCVcc: Cell culture grown HCV
qRT-PCR: Quantitative real-time PCR
PGSM: *Platycodon grandiflorum* saponin mixture
RdRp: RNA-dependent RNA polymerase
DAA: Direct acting antiviral.
